# Calcineurin-independent NFATc1 signaling is essential for survival of Burkitt lymphoma cells

**DOI:** 10.3389/fonc.2023.1205788

**Published:** 2023-07-21

**Authors:** Krisna Murti, Hendrik Fender, Carolin Glatzle, Rhoda Wismer, Salvador Sampere-Birlanga, Vanessa Wild, Khalid Muhammad, Andreas Rosenwald, Edgar Serfling, Andris Avots

**Affiliations:** Department of Molecular Pathology, Institute of Pathology, and Comprehensive Cancer Center Mainfranken, Julius-Maximilians University of Wuerzburg, Wuerzburg, Germany

**Keywords:** apoptosis, Burkitt lymphoma, cyclosporin A, nuclear localization, NFATc1, activated B cell-like diffuse large B-cell lymphoma (ABC-DLBCL), B cell receptor (BCR), Burkitt lymphoma (BL)

## Abstract

In Burkitt lymphoma (BL), a tumor of germinal center B cells, the pro-apoptotic properties of MYC are controlled by tonic B cell receptor (BCR) signals. Since BL cells do not exhibit constitutive NF-*κ*B activity, we hypothesized that anti-apoptotic NFATc1 proteins provide a major transcriptional survival signal in BL. Here we show that post-transcriptional mechanisms are responsible for the calcineurin (CN) independent constitutive nuclear over-expression of NFATc1 in BL and *Eµ-MYC* – induced B cell lymphomas (BCL). Conditional inactivation of the *Nfatc1* gene in B cells of *Eµ-MYC* mice leads to apoptosis of BCL cells *in vivo* and *ex vivo*. Inhibition of BCR/SYK/BTK/PI3K signals in BL cells results in cytosolic re-location of NFATc1 and apoptosis. Therefore, NFATc1 activity is an integrated part of tonic BCR signaling and an alternative target for therapeutic intervention in BL.

## Introduction

Burkitt lymphoma (BL) is an aggressive germinal center (GC) derived B cell lymphoma that is specified by *IgH-MYC* translocations, a high level of MYC expression and specific gene expression profile (1–4). For survival, BL cells need to counteract the apoptotic activity of MYC overexpression (5).

Several lines of evidence indicate that the majority of B cell lymphomas relay on B cell receptor (BCR) signaling for survival [reviewed in (6)]. In BL, translocation of *MYC* gene occurs exclusively to the nonproductively rearranged *IgH* locus. Several transgenic (tg) mouse models suggested and proved an important role of BCR signaling in MYC-driven lymphomagenesis (7, 8). In human BL, augmented BCR signaling were linked to the TCF3/ID3-mediated activation of pro-survival phosphatidylinositol-3 kinase (PI3K) pathway (9).

Activated B cell-like diffuse large B-cell lymphomas (ABC-DLBCL) depend on ‘chronic active’ BCR signaling which, similar to antigen (Ag) - dependent activation of normal B lymphocytes, engage multiple signaling cascades (10), as the MAPK, PI3K, NF-*κ*B and NFAT networks. In contrast, the survival of BL cells depends on antigen-independent ‘tonic’ BCR signaling which engages only the PI3K pathway (6, 11, 12). And indeed, loss of *Nfkb2* gene accelerated B cell lymphoma (BCL) development in *Eμ-MYC* tg mice (13), whereas the *Nfkb1* gene was shown to be dispensable for MYC-induced lymphomagenesis (14), and overall NF-*κ*B (15) and Calcineurin (CN)–dependent NFATc2 activation (16) induced apoptosis in human BL cells.

We and others (17, 18) have shown that NFATc1 proteins are expressed in most of human B-cell neoplasms. The family of NFAT transcription factors consists of the four closely related NFATc1, c2, c3 and c4 members, and the distantly related NFAT5 protein (19). Transcription of the *Nfatc1* gene in lymphocytes is under control of the two alternative promoters *P1* and *P2*, which together with immune receptor-inducible termination/polyadenylation signals give rise of six NFATc1 protein isoforms with individual properties. In B and T lymphocytes, NFATc1 expression is strictly controlled by Ca^2+^/CN signals that can be inhibited by the immune suppressants cyclosporin A (CsA) and FK506. Immune receptor triggering induces the massive synthesis of short NFATc1/αA isoform which counteracts the activation induced cell death (AICD) (20). The longer NFATc1/B and/C isoforms exert rather pro-apoptotic properties (21). However, in cells of myeloid lineage, NFATc1 is expressed as a near equal mix of NFATc1A, -B and -C proteins (22) suggesting that apoptotic properties of NFATc1 isoforms might be cell-type specific.

We will show here that in BL and BCL cells the nuclear expression of all NFATc1 protein isoforms provides a strong, Ca^2+^-dependent but CN-independent anti-apoptotic signal. Inhibition of ‘tonic’ BCR signaling in BL cells leads to cytosolic relocation of NFATc1, and gallium-mediated down-regulation of NFATc1 expression results in apoptosis of BL cells. Therefore, NFATc1 activity is an integrated component of tonic BCR signaling that is ‘hijacked’ from classical BCR signaling and should be considered as an alternative target for therapeutic intervention in BL cells.

## Materials and methods

### Mice

All animal experiments were conducted according to project licenses (Nr.55.2-2531.01-80/10 and 169), which were approved by the Regierung von Unterfranken, Würzburg. The following mice lines were used: C57BL/6 WT (Jackson Laboratory/Charles River), B6.*Eµ-myc* (23), *B6.Nfatc1^flx/flx^* (24) and B6.mb1-cre that have been described previously (25). All animals used were housed in the central animal facility (ZEMM) of University of Würzburg following standard animal care procedures. In each experiment, age-matched mice were used without any gender bias.

### Isolation and culture of B cells

Naïve splenic B cells were isolated using the Miltenyi Biotec kit #130-090- 862 for the isolation of mouse B cells. Primary B cells were cultured in X-vivo 15 medium, supplemented with 10% FCS (v/v), 2 mM L-glutamine, 100 μM NEAA, 1 mM Na-pyruvate, 100 U/ml penicillin/streptomycin and 50 μM β-mercaptoethanol (Gibco). In some assays, B cells were stimulated with TPA [(T; 10 ng/ml) 12-0 tetradecanoyylphorbol-13-acetate, Sigma-Aldrich], and ionomycin (I; 0.5 μM; Merck) with and without CsA (100 ng/ml). Then, they were incubated in RPMI-1640 medium supplemented with 10% heat-inactivated FCS, 1% chicken serum, 2-mercaptoethanol (50 μM), and L-glutamine (2 mM) at 37°C in 5% CO_2._

### Culture of human cell lines

The human BL cell lines Ramos, Namalwa, DND39, and Daudi, and Balm14, Balm31, BV-173, and the P-493-6 human B cell line immortalized with EBV were cultivated in RPMI, supplemented with 10% FCS and 0.1% β-mercaptoethanol.

### Immunohistochemistry of human BL samples and cells

Formalin-fixed and paraffin-embedded samples from 22 primary human BL cases and from tonsils were obtained from the histopathology files of the Pathology Institute, University Wuerzburg. When biopsy material was studied, the permission and consent of patients was received before. Immune stains of paraffin slices were performed with Abs directed against NFATc1 (clone 7A6, BD Pharmingen) and NFATc1/α (anti-IG-457). All pictures were captured with an Olympus Color view camera mounted on an Olympus BX41 dual-head light microscope. The pictures were taken with a Leica Confocal Laser Scanning Microscope (TCS SP5 II) and were analyzed with the Leica Software Image Pro Plus. For further demonstration, the digital images were processed using Adobe Photoshop CS3, Irfan view or Microsoft Office Power Point 2010.

For confocal microscopy, Namalwa and Ramos cells, *Eµ-MYC* induced mouse primary tumor cells,(#1542T), B cell lymphoma (BCL) cells from the same tumor (#1542B) and naïve splenic B cells were incubated for 1 h with αNFATc1 (7A6), αNFATc1/α (anti-IG-457) and, in some assays, αKi-67. Next, they were stained by secondary fluorescent labelled goat-anti-mouse Alexa Fluor (AF) 488, or goat-anti-rabbit AF 555, or AF 488, or by strepavidin AF 488 (all from eBiosciences), or goat-anti-mouse AF 647 (Dianova), or donkey anti-goat AF 488 (Invitrogen).

### Luciferase assays

Ramos B and Jurkat T cells were transfected with luciferase reporter constructs driven either by a minimal promoter alone (TATA-Luci) or by additional 3 copies of distal NFAT-binding site from the murine *Il2 promoter* (3xNFAT-Luci), together with an eGFP expression vector. After 12 h, the cells were induced with T+I with or without CsA. Luciferase activities were determined 12 h later and normalized to eGFP expression that was determined by flow cytometry.

### TUNEL assay and quantitation of apoptotic cells

The selected paraffin block of *Eµ-MYC* induced BCL tumors and tumors induced in littermate *Eµ-MYC^+^x Nfatc1^flx/flx^ x mb1-cre^-^* and *-mb1-cre^+^* mice were sectioned in 3 μm slices, followed by de-paraffinization and rehydration. Afterward, the tissues were permeabilized using proteinase K. Endogenous peroxidase was inactivated by 3% H_2_O_2_ and equilibrated by 1X reaction buffer.

### Immunoblotting

Western blot assays were performed by fractionating either whole or cytoplasmic and nuclear proteins on PAGE-SDS gels followed by detection of NFATc1 using the 7A6 mAb or NFATc1/α Ab (IG-457). In addition, the expression of BCL6 (using Ab D65C10, Santa Cruz), c-Myc (9E10, Santa Cruz), caspase-3, caspase-7 (Santa Cruz) and NFATc2 (Santa Cruz) was investigated. In other assays, Namalwa cells were cultivated either with or without GaN treatment in the presence SYK inhibitor (P505-15) to detect p-AKT, AKT, p-mTOR, mTOR, and p-S6 proteins (all Abs from Santa Cruz). Signals were developed using a chemiluminescence detection system (Thermo Fisher Scientific).

### RT-PCR assays

RNA was extracted from tumors of BL patients, BL cell lines, *Eµ-Myc* tumor cells, *Eµ-Myc* secondary tumors and resting splenic B-cells of WT mice using TRIzol reagent (Invitrogen), and RT-PCR assays were performed with primer sequences for detecting *Nfatc1 P1 and P2* promoter directed transcripts.

Semi-quantitative RT-PCR assays were performed to estimate the expression levels of the indicated genes (*NFATc1 mRNA*) in normal CD19^+^ human B-cells, human BL tumor and Ramos cells. Samples were normalized to the expression of *ACTB mRNA*. Further semi-quantitative RT-PCR assays were conducted to determine the *NFATc1 P1 and P2 promoter* activities in human BL, Ramos and Namalwa cells. The primer sequences are presented in the supplement.

### Measurement of intracellular Ca^2+^ level

Ramos cells and #1542B BCL cells were treated with the cell-permeable Ca^2+^ chelator BAPTA AM [(10 µM) 1,2-Bis(2-aminophenoxy) ethane; Santa Cruz Biotechnology]; or CsA (0.5 µg/ml) for 4 h. To assess the expansion of Ramos and #1542B BCL cells, BAPTA AM was added in concentrations of 5, 10, 20 and 40 µM, and DMSO was used as a control. Half of the medium (including inhibitors) was renewed daily.

### Apoptosis assays and flow cytometry

Apoptosis assays were conducted with tumors cells of *Eµ-MYC* tg mice with ´Small Pre-B’, ´Immature B´, ´Mature B´ and ´Mixed´ immunophenotypes. 5 × 10^5^ cells were stained with annexin V APC (BD Pharmingen). The samples were measured within 1 h after staining and 1 μl of PI (1mg/ml) was added just before the measurement. Flow cytometry and data analysis were performed following standard procedures using the FACS calibur and FlowJo software.

### Co-culture experiments

*Eµ-Myc* tumor cells (#0435) were co-cultured on 40LB feeder cells that express the CD40-ligand (and BAFF (26)) in the presence and absence of IL-4 for 4 d.

### Determination of Myc activity on NFATc1 stability

Human lymphoblastoid P493-6 cells were cultivated for 3 d without treatment (MYC on) and treated with doxycycline (0.1 µg/ml, MYC ‘off’), or 17β-estradiol (1 mM; MYC ‘on’ and EBNA ‘on’), or both doxycycline and 17β-estradiol (MYC ‘off’ and EBNA ‘on’), followed by Western blots of nuclear and cytosolic proteins which were performed with Abs directed against MYC, NFATc1 and NFATc2. To measure half-life of NFATc1 in additional assays in the presence of MYC, P493-6 cells were cultivated in normal medium (MYC ‘on’) or in the presence of doxycycline and 17β-estradiol (MYC ‘off’) for 3 d followed by treatment with CHX [(250 µg/ml); cycloheximide, Sigma Aldrich]. Western blots were performed with Ab directed against NFATc1 (7A6), and the relative NFATc1 protein levels were estimated by densitometry.

### Statistical analysis

Student t tests were employed to assess the experimental data by using GrapPad Prism version 5 (GrapPad Software Inc., California). P values ≤ 0.05 were considered as statistically significant.

## Results

### Predominant nuclear localization of NFATc1 and NFAT-dependent transcriptional activity in BL

**Figure 1:**
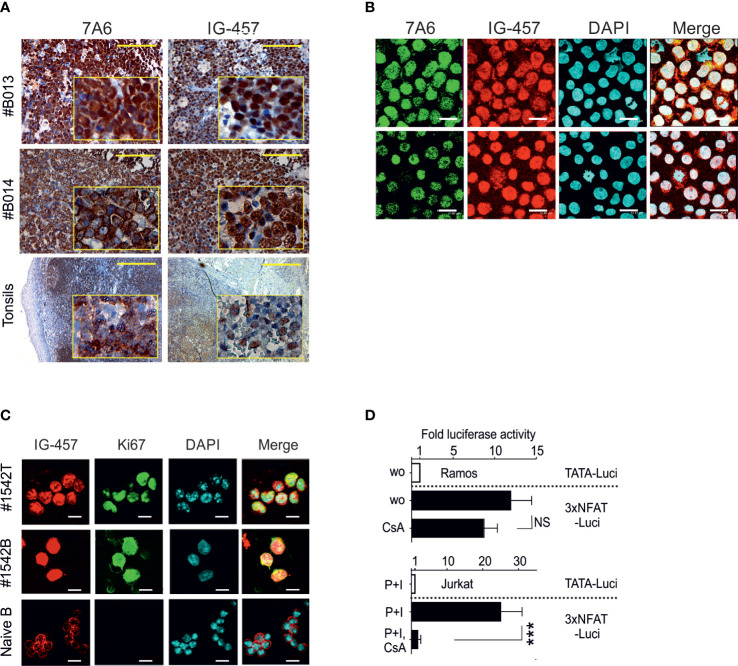
Predominant nuclear localization of NFATc1 in BL cells. **(A)** Representative immunohistochemical stains of human primary BL cells (n=22, see also **Figure S1**) with an Ab directed against all NFATc1 (7A6) or NFATc1/α (IG-457) proteins, compared to normal human tonsils (lower panel). Scale: 400 μm. **(B)** Confocal microscopy of NFATc1 expression in Ramos (upper panel) and Namalwa BL (lower panel) cells (see also **Figure S2**). Scale: 16 μm. **(C)** Confocal microscopy of NFATc1 expression in a *Eµ-MYC* induced mouse primary tumor (#1542T), in a BCL cell line derived from the same tumor (#1542B) and in naïve splenic B cells using Abs directed against NFATc1/α and Ki67. Scale: 13 μm. **(D)** Ramos and Jurkat cells were transfected with luciferase reporter constructs driven either by a minimal promoter (TATA-Luci) or by additional 3 copies of distal NFAT-binding site from the murine *Il2* promoter (3xNFAT-Luci), together with an eGFP expression vector. After 12 h the cells were induced with TPA (T, 10 ng/ml), Ionomycin (I, 50 μM) or CsA (0.1 μg/ml), as indicated. Luciferase activities were determined 12 h later and normalized to the eGFP expression determined by flow cytometry. Data shown are representative of 3 independent transfections and shown as the mean +SD. Statistical significance was determined by using unpaired Student’s *t*-tests. ***P ≤ 0.001.

To investigate the role of NFATc1 in BL we stained BL tumors with an Ab raised against all NFATc1 isoforms (Ab 7A6) or an Ab which reacts specifically with the NFATc1/α isoforms (Ab IG-457) (27, 28). In 21 from 22 analyzed tumors (**Figures 1A**, **S1**) we observed a strong overall expression of NFATc1 and of NFATc1/α isoforms, in contrast to tonsillar B-cells that showed a very mild background stain (with the exception of some germinal center-like spots: **Figure 1A**). In one case (**Figure S1**, ´Group C´), NFATc1 appeared overexpressed only in a fraction of tumor cells. In respect to subcellular localization, we observed two distinct staining patterns: an exclusive nuclear staining (14 tumors, ´Group A´, **Figures 1A**, **S1**) and a mixed nuclear/cytosolic staining (´Group B´, **Figure S1**). Both Abs revealed similar staining patterns suggesting the expression of both NFATc1/α and/β isoforms in BL tumors. In BL cell lines, NFATc1 was also predominantly expressed in nuclei (**Figures 1B**, **S2**), as well as in *Eμ-MYC*-induced primary BCL tumors (23) and derived cell lines (**Figure 1C**). Transient transfections of BL Ramos cells and Jurkat T cells with NFAT-luciferase reporter constructs indicated a strong constitutive NFAT-mediated activity on transcription in Ramos cells that remained almost unaffected by CsA. In contrast, the NFAT-mediated induction in Jurkat cells was completely suppressed by CsA (**Figure 1D**). These data show that the constitutive nuclear, CsA-insensitive expression of NFATc1 is a hallmark of BL tumor cells.

### NFATc1 suppresses apoptosis in *E*μ*-MYC* induced BCL tumors

To identify a role for NFATc1 in MYC-induced tumorigenesis, we investigated *Eμ-MYC* tg mice (23). Tumors that develop in these mice represent different stages of B-cell development (**Figures S3A, B**) and, according to our analyses of *Ig-H* gene rearrangements, consist of 5-8 individual clones (data not shown). As in human BL cells, activation of NF-*κ*B pathway by CD40LB feeder cells (26) induces apoptosis in these BCL cells (**Figure S3C**) (13–15).

**Figure 2:**
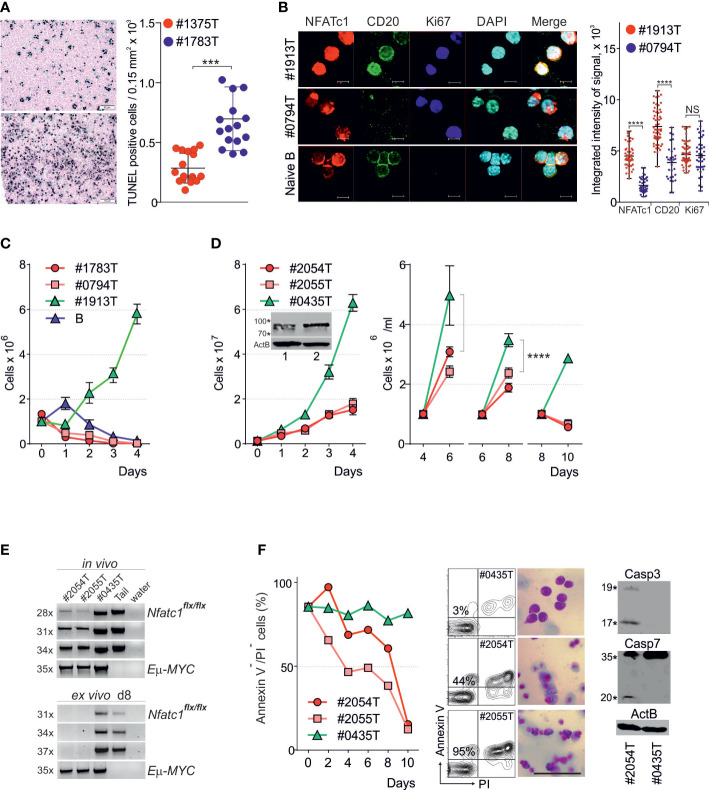
NFATc1 suppresses apoptosis in *Eμ-MYC* induced BCL tumors **(A)** Representative TUNEL assay (left) and quantitation of apoptotic cells (right) in tumors induced in littermate *Eμ-MYC^+^x Nfatc1^flx/flx^x mb1-cre^-^* (above; #1375T) and *-mb1-cre^+^* mice (below; #1783T). **(B)** Representative confocal microscopy analyses (left) and quantitation of NFATc1 (7A6), CD20 and Ki67 expression in freshly isolated primary tumor cells from *Eμ-MYC^+^ x Nfatc1^flx/flx^ x mb1-cre^-^* (#1913P) and *-mb1-cre^+-^* mice (#0794P), in comparison with naïve B-cells isolated from WT mice. Scale: 15 mm. **(C)** *Ex vivo* expansion of primary tumor cells from *Eμ-MYC^+^ x Nfatc1^flx/flx^ x mb1-cre^+^* (#1783T, #0794T) and *-mb1-cre^-^* (#1913T) mice, in comparison with anti-IgM stimulated naïve B cells isolated from WT mice **(B)**. **(D)** *Ex vivo* expansion of tumor cells from *Eμ-MYC^+^ x Nfatc1^flx/flx^ x mb1-cre^-^* (#0435T) and tumors from *-mb1-cre^+^mice* (#2054T and #2054T, respectively). After initial expansion (left panel) the cells were split on d 4, d 6 and d 8 (right panel). The insert shows Western blot assays of NFATc1 expression on d 3 in #2054T (1) and #0435T (2) tumor cells. **(E)** PCR analyses of *Nfatc1^flx/flx^* and *Eμ-MYC* alleles in the indicated tumors *in vivo* (above) and after 8 d of cultivation *ex vivo* (below). A biopsy from tumor-free *Nfatc1^flx/flx^* mice (Tail) was used as control. **(F)** Survival of tumor cells during *ex vivo* cultures was analyzed by annexin V and PI staining (left). Middle and right – representative flow cytometry analyses, haematoxylin-eosin staining and Western blot analyses of d 8 cultures using Abs directed against activated caspase-3 and both forms of caspase-7. Statistical significance was determined by using unpaired Student’s t-tests. ***P ≤ 0.001; ****P ≤ 0.0001. ns, statistically non-significant.

For the conditional inactivation of *Nfatc1* gene in B-cell lineage we crossed *Eμ-MYC* tg mice with *Nfatc1^flx/flx^* x *mb1-cre* mice (25). The survival of *Eμ-MYC x Nfatc1^flx/flx^ x mb1-Cre^+^* mice remained unaffected (data not shown). However, the tumors in those mice (#1783T) were specified by high numbers of apoptotic tumor cells (**Figure 2A**) and increased numbers of tumor infiltrating macrophages (not shown). Confocal microscopy indicated that these tumor cells still expressed NFATc1 proteins (**Figures 2B, D**), although at reduced levels and in part in cytosol. Importantly, like unstimulated naïve B cells, these cells were also specified by partial granulation of nuclei suggesting the induction of apoptosis. Genotyping confirmed the presence of an ‘intact’ *Nfatc1^flx^* allele in these tumor cells (**Figure 2E**), which might be due to the limited activity of *mb-1* - directed Cre-recombinase *in vivo*. All tested *Nfatc1^flx/flx^x mb1-Cre^-^* tumors vigorously proliferated *ex vivo* (as shown for the tumor #1913 T in **Figure 2C**; n=17) whereas the *Nfatc1^flx/flx^ x mb1-Cre^+^* tumors (as #1783T and #0794T) either did not proliferate (n=5) or proliferated slowly (n=2) (**Figure 2D**, left panel). During further passages (**Figure 2D**, right panel) such cultures (#2054T and #2055T) lost the ‘intact’ *Nfatc1^flx^* allele (**Figure 2E**), ceased to proliferate and underwent apoptosis (**Figure 2F**). This is shown in **Figure 2F** for the NFATc1^-/-^ clones #2054T and #2055T by Annexin V and PI stains and caspase 3/7 cleavage (c#2054). In contrast, upon culture for 8-10 d, the cells of clone #0435 expressed still NFATc1, proliferated vigorously and did not show an increase in apoptosis (**Figure 2F**),

These data indicate that NFATc1 expression is required for the survival of BCL cells ex vivo.

### NFATc1 nuclear localization and expansion of BL cells depend on intracellular Ca^2+^

**Figure 3:**
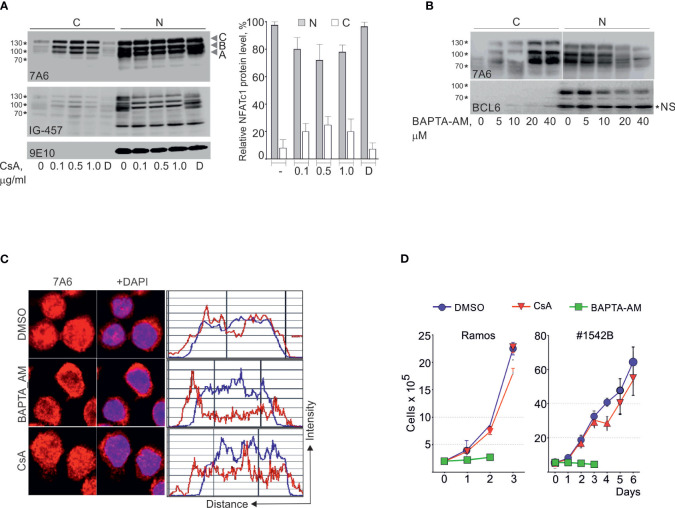
NFATc1 nuclear localization and expansion of BL cells depend on intracellular Ca^++^ level but not CN activity. Ramos cells were cultivated in the presence of the indicated concentrations of CsA **(A)** or BAPTA-AM, a cell-permeable Ca^2+^ chelator **(B)** for 4 h followed by Western blots of nuclear (N) and cytosolic **(C)** protein fractions using Abs directed against NFATc1 (7A6), NFATc1α (IG-457), BCL6 and MYC (9E10). The positions of NFATc1/A,/B and/C isoforms are indicated. Quantitation of NFATc1 protein levels in nuclear and cytosolic fractions is shown in the right panel. **(C)** Confocal microscopy analyses of NFATc1 localization in the Ramos cells treated with BAPTA-AM (10 μM) or CsA (0.5 μg/ml). Scale: 16 μm. **(D)** Expansion of Ramos and #1542B BCL cells in the presence of CsA (0.5 μg/ml) or BAPTA-AM (5 μM). Half of the medium (with inhibitors) was renewed daily. Data are representative of 3 individual experiments. NS, non-specific band.

In contrast to expression in activated lymphocytes (27), in Ramos and Namalwa BL cells NFATc1 is expressed in nuclei as an equimolar mix of NFATc1A, -B and –C isoforms, each with alternative α- or β- N-terminal peptides (**Figure 3A**). Treatment of Ramos or Namalwa cells with CsA resulted in a phosphorylation of nuclear NFATc1 (notice the mobility shift of nuclear NFATc1 protein bands in **Figure 3A**), but only a minor fraction of nuclear NFATc1 was re-located to the cytosol (**Figure 3A**, right panel and **Figure 3C**). The same treatment led to the complete relocation of RELA protein to the cytosol in Ramos cells (**Figure S2C**). In addition, CsA treatment did not affect the high proliferation rate of BL or BCL cells (**Figure 3D**) whereas treatment of the cells with the cell-permeable Ca^2+^ chelator BAPTA-AM reduced strongly the nuclear NFATc1 fraction (**Figures 3B, C**), suppressed proliferation (**Figure 3D**) and induced the apoptosis in Ramos and BCL cells (**Figure 3D**). These findings suggest that Ca^2+^ - dependent NFATc1 activity is essential for the survival of BL cells.

### Post-transcriptional control of NFATc1 expression in human BL cells

**Figure 4:**
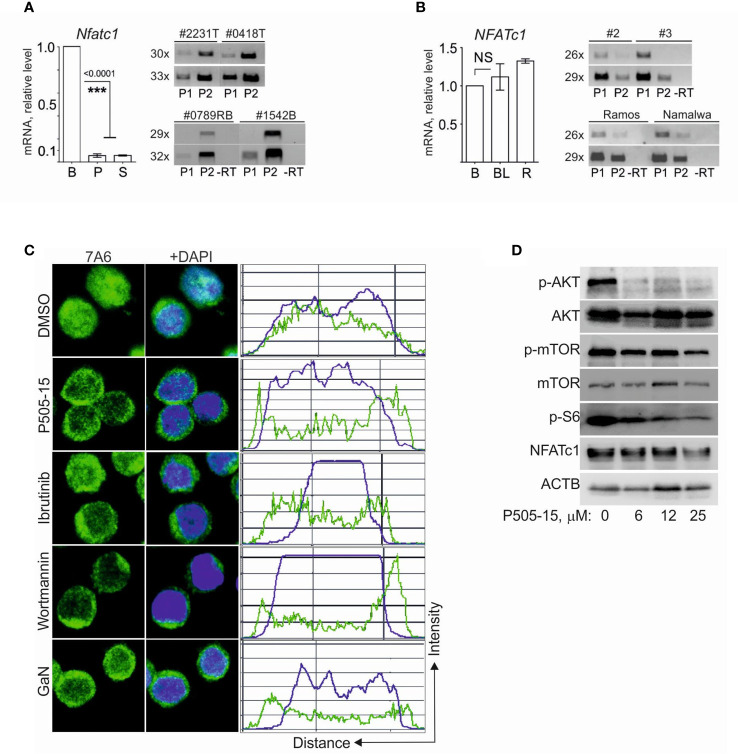
NFATc1 expression in BL and in BCL cells is regulated at the post-transcriptional level. **(A)** Left, analyses of *Nfatc1* mRNA expression in naïve murine B cells **(B)** and BCL cells, purified from primary (P, n=3) or secondary (S, n=2) BCL tumor cells. Samples were normalized to expression of *ActB* mRNA. Right, semi-quantitative RT-PCR analyses of *Nfatc1 P1* and *P2* promoter activities in primary BCL tumors (#2231T and #0418T, above) and in established BCL cell lines (#0789RB and #1542B, below). **(B)** Left, real-time PCR analyses of *NFATC1* mRNA expression in normal CD19^+^ human B-cells **(B)**, in human tumor specimens (BL, n=4) and in Ramos cells (R). Samples were normalized to the expression of *ACTB* mRNA. Right, semi-quantitative RT-PCR analyses of *NFATC1 P1* and *P2* promoter activities in human BL specimens (#2 and #3, above) and in Ramos and Namalwa cells (below). **(C)** Confocal microscopy of subcellular localization of NFATc1 in Namalwa cells cultivated in the presence of P505-15 (20 μM), ibrutinib (20 μM) or wortmannin (1 μM) for 4 hr or GaN (0.75 mM) for 20 h. **(D)** Immune blots of whole cell protein extracts from Namalwa cells that were cultivated in the presence of indicated concentrations of P505-15 for 4 h. The blots were performed with general and phospho-specific Abs directed against the proteins indicated. Statistical significance was determined by using unpaired Student’s t-tests. ***P ≤ 0.001. ns, statistically non-significant.

Analysis of Gene Expression Omnibus (29) datasets (GSE26918) revealed a strong reduction of *Nfatc1* mRNA levels in pre-malignant B-cells from *IgΛ-MYC* tg mice (30) (**Figure S4**). We observed a similar decrease in NFATc1 RNA levels in primary and secondary BCL tumors from Eμ-Myc mice (**Figure 4A**, left panel), compared to primary B cells. In those tumors, the majority of *Nfatc1* transcripts was directed by the *P2* promoter (**Figure 4A**, right panel) and therefore represents NFATc1/β isoforms. Surprisingly, in human BL and Ramos tumor cells the expression level of *NFATc1* mRNA was as high as in primary human B cells (**Figure 4B**, left panel), and the majority of *NFATc1* transcripts was driven by the *P1* promoter. This means that in human BL cells NFATc1/α proteins are predominantly generated (31) (**Figure 4B**, right panel).

Tonic BCR signaling through the PI3K signaling cascade is a molecular property of BL cells (6). When we suppressed the signaling transfer through this cascade by inhibitors of SYK, BTK and PI3 kinases, i.e. by P505-15, ibrutinib and wortmannin, respectively, in Nawalma tumor cells, we observed the relocation of nuclear NFATc1 into cytosol (**Figure 4C**). While the SYK inhibitor P505-15 suppressed the phosphorylation of AKT and ribosomal protein S6, and impaired mTOR phosphorylation, it did not affect the overall expression of NFATc1 in Nawalma cells (**Figure 4D**). This shows that in human BL cells the expression of NFATc1 is predominantly controlled at the post- but not transcriptional level.

A similar conclusion can be drawn from the effect of Gallium nitrate (GaN) on BL cells, a compound that is known to down-regulate NFATc1 expression in osteoclasts (32). While GaN treatment led to the re-localization of nuclear NFATc1 to the cytosol (**Figure 4C**), it did not inhibit the overall NFATc1 expression within 24 h (albeit mildly in high concentrations within 3-4 d, which affected also the proliferation and survival of cells [**Figure S5**]).

**Figure 5:**
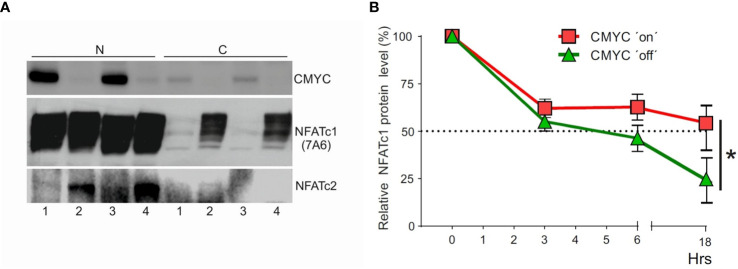
Effect of Myc expression on the localization and stability of NFATc1. **(A)** Human lymphoblastoid P493-6 cells were cultivated for 3 d without treatment (1) or treated with doxycycline (0.1 μg/ml, lane 2), 17β-estradiol (1 mM, lane 3), or both doxycycline and 17β-estradiol (lane 4). Western blot assays of nuclear (N) and cytosolic C protein extracts were performed with Abs directed against MYC, NFATc1 and NFATc2. **(B)** P493-6 cells were cultivated in normal medium (MYC ‘on’) or in the presence of doxycycline and 17β-estradiol (MYC ‘off’) for 3 d followed by treatment with CHX (250 μg/ml) for 18 h. Western blots were performed with Abs directed against NFATc1 (7A6), and the relative NFATc1 protein levels were estimated by densitometry. The results of one representative assay (from three) are shown. The results of three assays revealed similar, albeit not identical effects of c-Myc on NFATc1 stability. The error bars reflect different exposure times of the same membrane. Statistical significance was determined by using unpaired Student’s t-tests. *P ≤ 0.05.

To elucidate a role of MYC in control of NFATc1 expression in human BL cells, we studied P493-6 BL cells (33). These cells are derived from human peripheral B cells after immortalization with EBNA2-ER, a fusion protein of nuclear EBV antigen and the estrogen receptor. And, in addition, these cells harbor a *MYC* transgene that can be repressed by doxycycline (33). When we suppressed the expression of MYC by doxycycline, we observed a remarkable increase in cytosolic NFATc1 (**Figure 5A**). The downregulation of MYC enhanced NFATc2 protein expression but did not affect the proliferation rate of P493-6 BL cells (not shown). In these BL cells, the expression of MYC increased the half-live of NFATc1 protein (5 h against 18 h, **Figure 5B**). These data indicate that high levels of MYC stabilizes NFATc1 and support the nuclear localization of NFATc1 in BL cells.

## Discussion

The results of our study show that the transcriptional activity of NFATc1 is critical for the survival of BL and *Eμ-MYC* - induced BCL tumors (**Figures 1**, **2**). Our initial incentive to investigate the role of NFATc1 in BL came from the observation that anti-apoptotic NFATc1/α proteins are expressed in a wide panel of lymphomas (17). As NF-κB signals suppress MYC – induced lymphomagenesis (15), we hypothesized that NFATc1 might be important to counteract the pro-apoptotic activity of high-level MYC expression in BL. And indeed, almost all BL that we investigated express nuclear NFATc1 (**Figures 1A-C**, **S1**), which therefore is another hallmark of BL.

We have shown before that the short anti-apoptotic NFATc1/αA isoform counteracts, and the longer NFATc1/B+C isoforms and NFATc2 promote apoptosis in T and B lymphocytes (21, 34, 35). BCR triggering induces the massive synthesis of a short, anti-apoptotic NFATc1/αA isoform in normal B cells. In contrast, in BL entities NFATc1 is expressed as a near equimolar mix of -A, -B and -C isoforms (**Figures 3A, B**). We observed previously a similar NFATc1 expression pattern in peritoneal resident macrophages (22) implying that anti- and pro-apoptotic properties of NFATc1 isoforms are cell-type specific. In human BL cells transcription of the *NFATc1* gene is mainly driven by the *P1* promoter, whereas *P2* promoter activity is dominant in mouse BCL tumors and derived cell lines (**Figures 4A, B**). This might reflect the different origin of human BLs, which arises from GC B cells where NFATc1/α isoforms are expressed (36), and murine BCL tumors that originate at earlier stages of B cell development (**Figures S3A, B**).

Despite intensive studies on gene expression profiles, NFATc1 was never identified before as an important survival factor in BL cells. This is not surprising, as *NFATc1* mRNA levels are not affected in human BL specimens (**Figure 4B**), and even strongly downregulated in *Eμ-MYC* (**Figure 4A**) and *Λ-MYC* (**Figure S4**) - induced tumors. We show that high-level nuclear expression/activity of NFATc1 in BL is achieved by post-transcriptional mechanisms (**Figures 4**, **5**). MYC expression not only drastically increases the half-life of NFATc1 protein (**Figure 5**) but through the increase of intracellular Ca^++^ levels (37) contributes to the nuclear localization of NFATc1 in P493-6 BL model. Interestingly, repression of MYC induces the expression of pro-apoptotic NFATc2 in these cells (**Figure 4C**).

In BL cells, the proliferation and nuclear localization of NFATc1 is not affected by the CN inhibitors CsA and FK506 (**Figures 3A**, **S2**). Both inhibitors induce the phosphorylation of NFATc1 proteins, but only a minor fraction of NFATc1 protein is relocated to the cytosol. Phospho-proteomic analyses of BL cells (11) did not reveal any tyrosine phosphorylation of NFATc1 and therefore ruled out the involvement of JAK - kinase dependent NFATc1 activation pathway (38).

Nucleocytoplasmic shuttling factors, which recognize nuclear export signal (NES) within the regulatory domain of NFATc1, are required for export of NFATc1 into the cytosol (39). It is likely that yet to be identified interacting protein masks NFATc1 NES in BL cells. We have shown before that NFATc1 facilitates activation of BCL6 – repressed chemokine genes in murine peritoneal resident macrophages (22). It remains to be shown if such transcriptionally active complex between NFATc1 and BCL6 does exist in BL cells and in GC B cells, from which human BL originate [reviewed in (40)].

Decreased intracellular Ca^++^ ([Ca^2+^]_I_) levels in BL cells induce NFATc1 relocation to cytosol and induces apoptosis (**Figure 3**). On the one hand, this is in agreement with the sustained increase in [Ca^2+^] in pre-malignant B cells from *Eμ-MYC* mice, where reduced Ca^2+^ efflux and constitutive NFAT nuclear translocation was observed (37). On the other hand, diminished release of Ca^2+^ and decreased Ca^2+^ influx in NFATc1^-/-^ B cells (35) suggest that NFATc1 itself plays an important role in the regulation of [Ca^2+^]_I_ in BL and in BCL cells. This is in line with the role of Ag-independent constitutive ‘tonic’ BCR signaling, which through the activation of the PI3K pathway ensures survival of BL and GCB DLBCL cells (6). Inactivation of a major ‘tonic’ BCR signaling mediators (SYK, BTK and PI3 kinases, **Figures 5A, B**), which regulate [Ca^2+^]_I_ in B cells (41–43) leads to the cytosolic relocation of NFATc1. The same is true for gallium-compounds, which affect NFATc1 nuclear translocation and expression level of NFATc1 (32, 44), without affecting the activity of PI3K pathway (**Figures 5C, D**). Therefore, NFATc1 activity is an integrated component of ‘tonic´ BCR signaling, which is ‘hijacked’ from classical BCR signaling pathway and should be considered as an alternative target for therapeutic intervention in BL and as diagnostic feature. Accordingly, gallium compounds, which are already in several phase II trials (45, 46), might be considered as an addition to the current regimens for treatment of BL and other cancers, where nuclear NFATc1 expression is evident.

Our study showed that the nuclear localization of NFATc1 is a molecular hallmark of BL cells. We observed the nuclear NFATc1 localization in (almost) all human BL samples, in human BL cell lines and in murine *Eμ-MYC* - induced BCL tumors. Therefore, it is likely that the various gene expression programs in BLs that are characterized by the expression of nuclear EBV antigens affect the nuclear expression of NFATc1. However, they might modulate the activity of nuclear NFATc1. This was shown recently by a comprehensive investigation of LMP2A activity on phosphorylation in BL cells that revealed a negative effect of serine/threonine phosphorylation on NFAT activity (47).

Taken together, our study revealed a novel molecular hallmark of human BL, i.e. the persistent nuclear accumulation of NFATc1 that plays an important role in the survival of BL cells. However, it remains to be shown at the molecular level which genes, which networks of signaling molecules are affected by NFATc1 that contribute to the survival of BL cells. This will need NGS, metabolomics and further extensive studies that could – along with comprehensive screenings – lead to candidate targets for a more causative immune therapies. Our findings presented here are a first step in this direction.

## Data availability statement

The original contributions presented in the study are included in the article/**Supplementary Material**. Further inquiries can be directed to the corresponding authors.

## Ethics statement

The animal study was reviewed and approved by project licenses (Nr.55.2-2531.01-80/10 and 169), which were approved by the Regierung von Unterfranken, Würzburg.

## Author contributions

KrM, HF and AA performed all experiments, with the help of RW and SS-B, VW and AR assisted with human BL specimens. KhM provided bioinformatic analyses. Together with ES, AA conceived the study, analyzed all experiments and wrote the paper. All authors contributed to the article and approved the submitted version.
