# Supplementary figures and images for: Calcineurin-independent NFATc1 signaling is essential for survival of Burkitt lymphoma cells
Source: Front Oncol. 2023 Jul 21;13:1205788. doi: 10.3389/fonc.2023.1205788 (PMC10403262; doi:10.3389/fonc.2023.1205788)

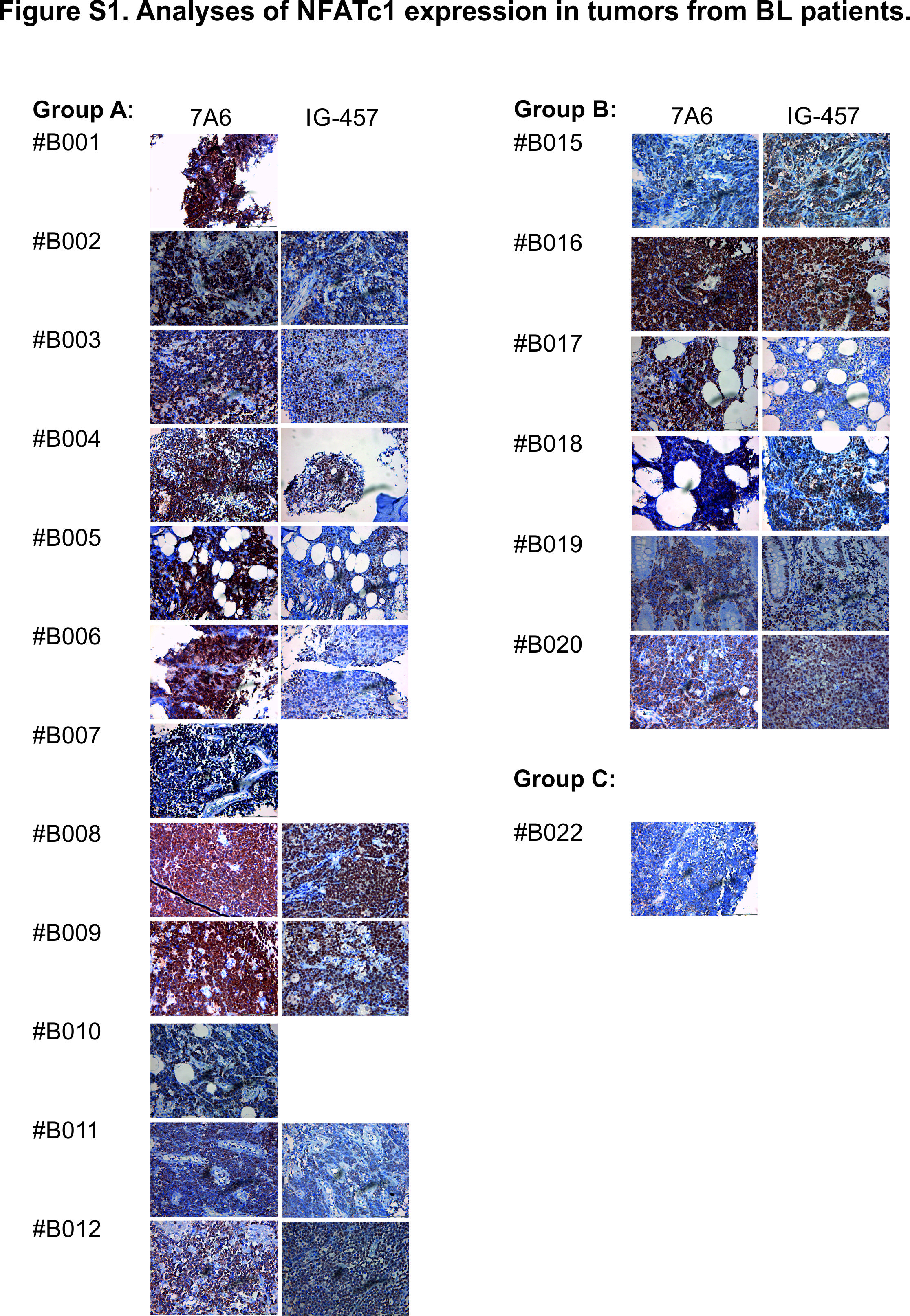

Supplement: Supplementary file 1 [file Image_1.jpeg]

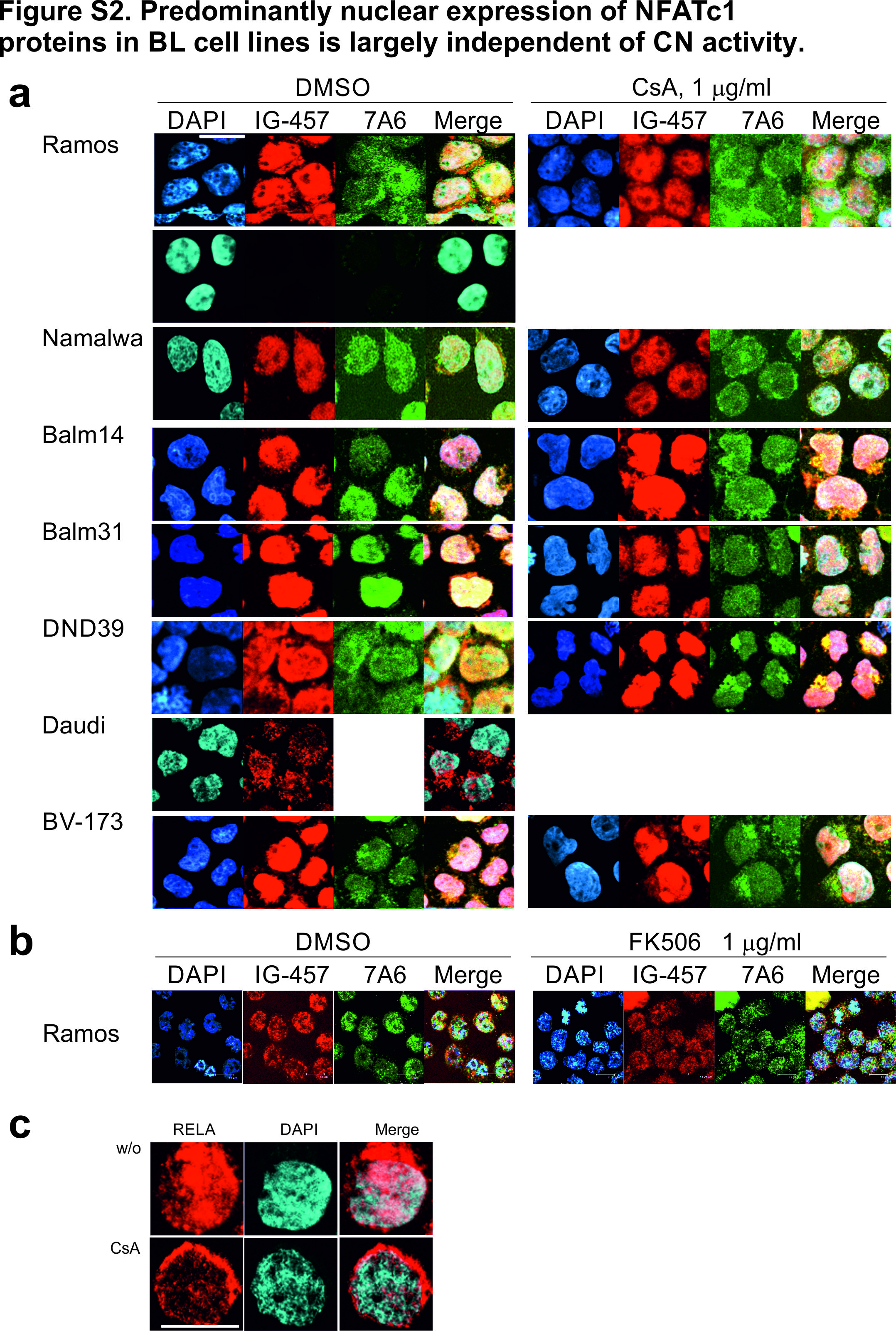

Supplement: Supplementary file 2 [file Image_2.jpeg]

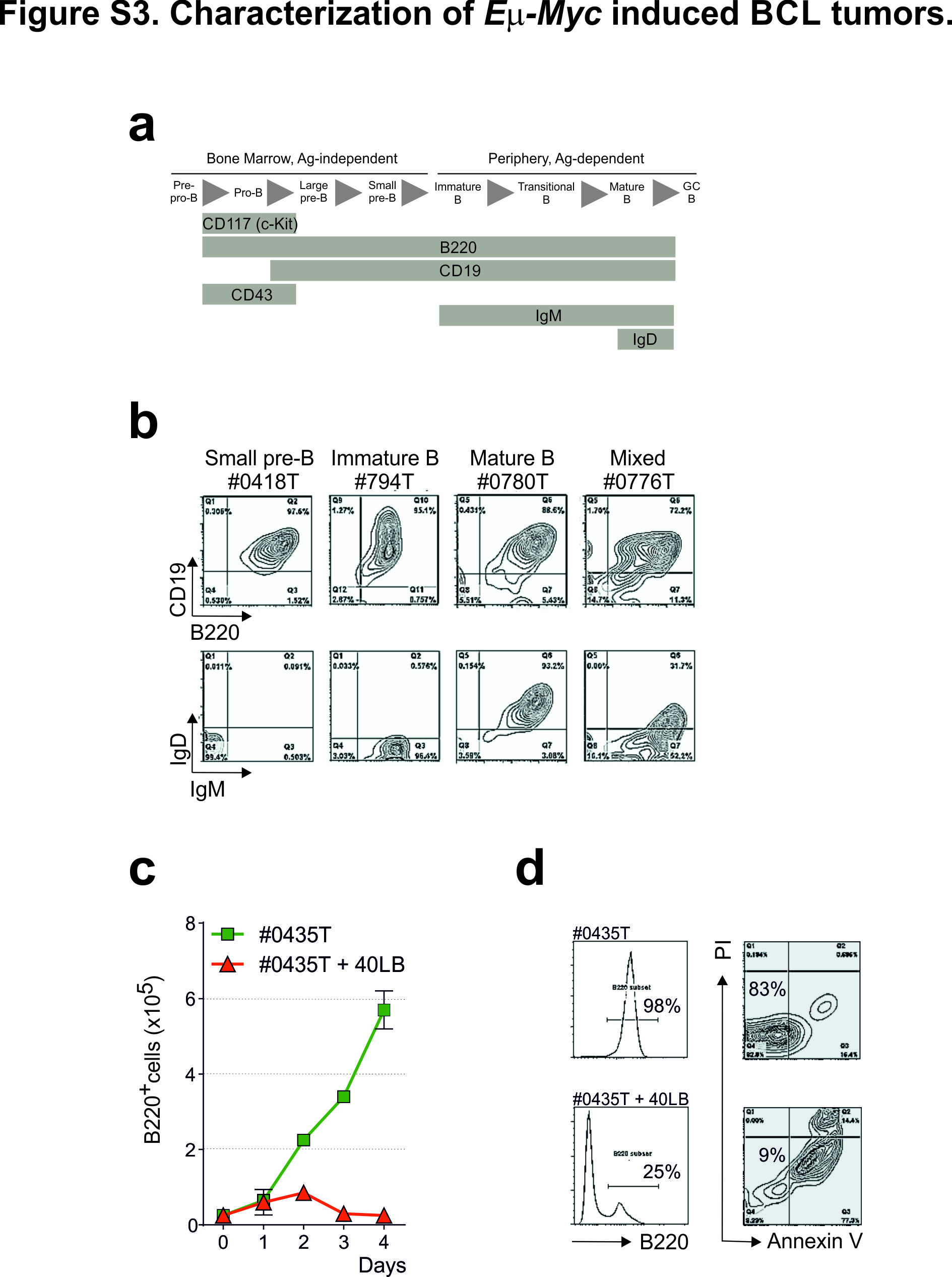

Supplement: Supplementary file 3 [file Image_3.jpeg]

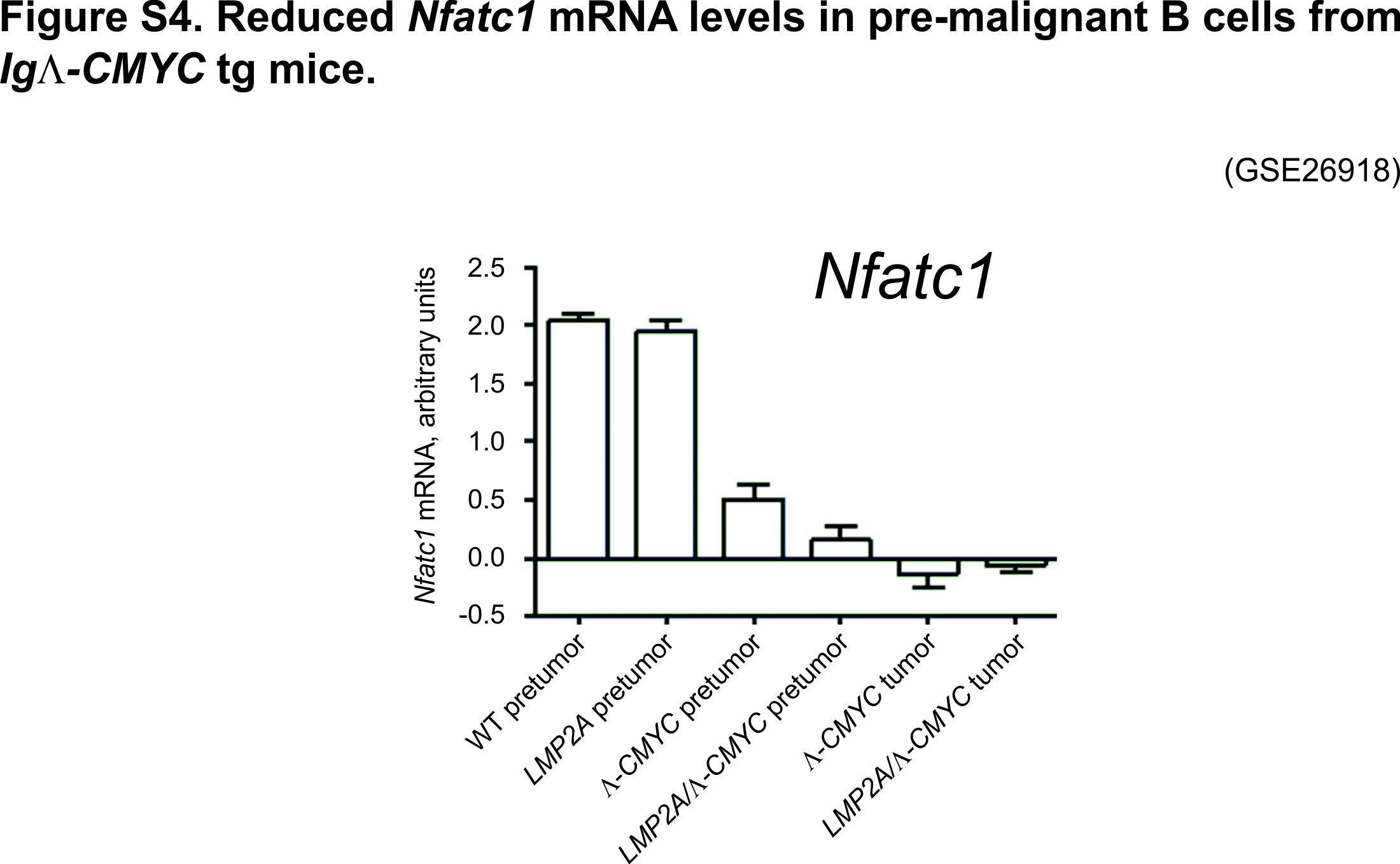

Supplement: Supplementary file 4 [file Image_4.jpeg]

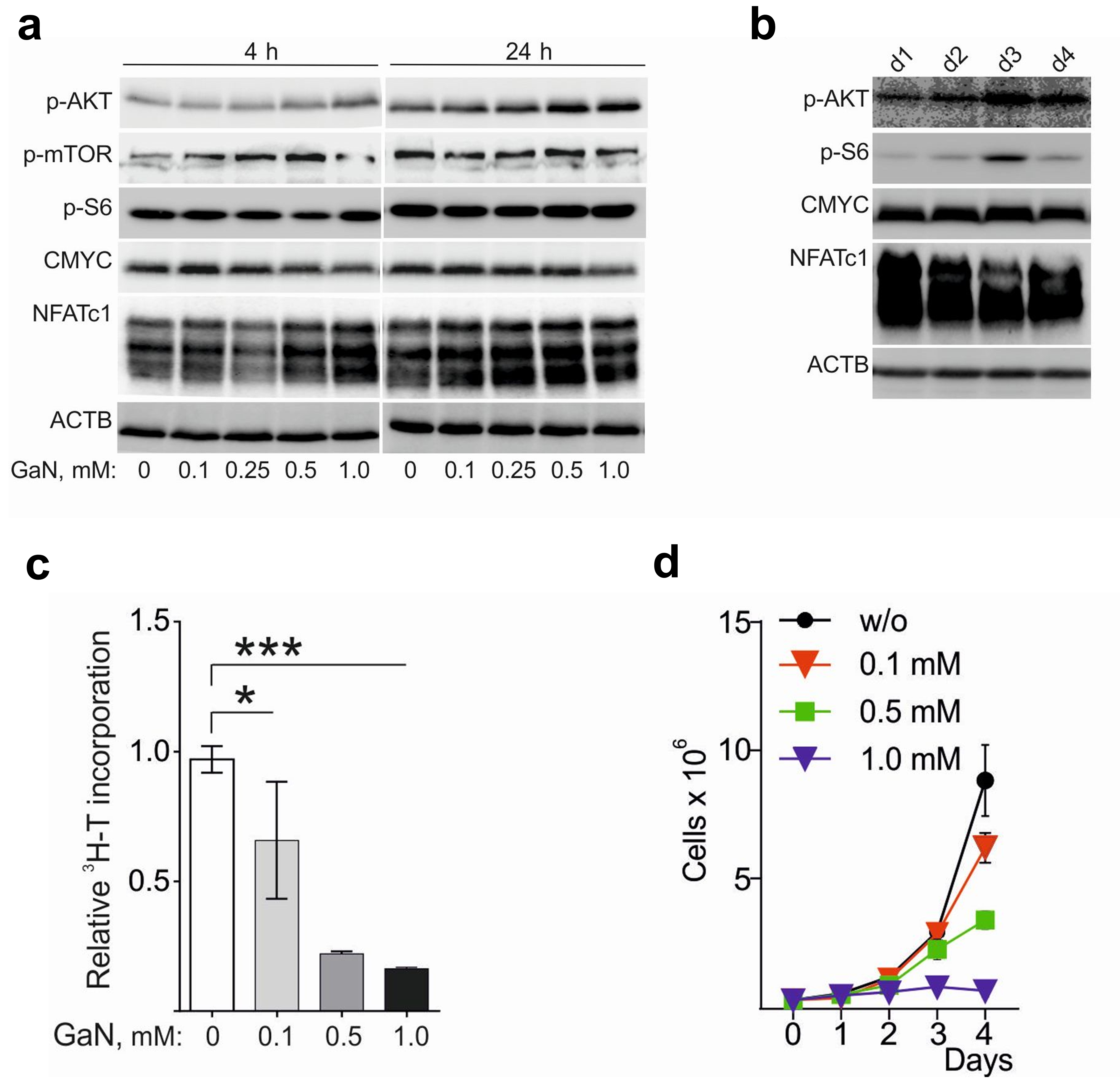

Supplement: Supplementary file 5 [file Image_5.jpg]
