# Supplementary material for: Calcineurin-independent NFATc1 signaling is essential for survival of Burkitt lymphoma cells
Source: Front Oncol. 2023 Jul 21;13:1205788. doi: 10.3389/fonc.2023.1205788 (PMC10403262; doi:10.3389/fonc.2023.1205788)
Supplement: Supplementary file 6 [file Table_1.pdf]

SUPPLEMENT

Figure S1. Analysis of NFATc1 expression in tumors from BL patients. Immune histochemical staining of human primary BL tumors with Abs directed against all NFATc1 proteins (7A6) or an NFATc1/α (IG-457) specific Ab. Definition of groups is based on staining intensity and NFATC1 sub-cellular localization. Group A - high intensity, predominant/exclusive nuclear staining. Group B - high intensity nuclear and cytosolic staining. Group C - weak intensity, predominant cytosolic staining. All images are at original 200x magnification, scale: 100 μm.

Figure S2. Predominant nuclear expression of NFATc1 protein in BL cell lines is independent on CN activity. (**a**, **b** and **c**) Indicated BL cell lines were incubated in the presence of solvent (DMSO), CsA (**a** and **c**, 1 μg/ml) or FK506 (**b**, 0.1 μg/ml) for 6 h and stained with Abs directed against NFATc1/α (IG-457), NFATc1 (7A6) or RELA, followed by confocal microscopy. As control, the cells were stained without primary Ab (**a**, Ramos, lower panel).

Figure S3. Characterization of *Eμ-MYC* induced BCL tumors. (**a**) Scheme of normal B-cell differentiation with selected cell surface markers indicated. (**b**) Flow cytometry of tumors from *Eμ-MYC* tg mice with ´Small Pre-B', ´Immature B´, ´Mature B´ and ´Mixed´ immune phenotypes. (**c** and **d**). Expansion of primary tumor (#0435T) cells alone or as a co-culture with 40 LB feeder cells. (**c**) Living tumor cells were enumerated daily. (**d**) After staining for B220 and PI/annexin V, the co-cultures were analyzed by flow cytometry on d 3.

Figure S4. Reduced *Nfatc1* mRNA levels in pre-malignant B cells from *IgΛ-MYC* Tg mice. Data from the *Gene Expression Omnibus* [29] dataset GSE26918 [30] are shown.

Figure S5. Effect of GaN treatment on BL cells. **(a+b)** Whole cell extracts were prepared from Namalwa cells cultivated in the presence of indicated concentrations of GaN for 4 or 24 h (a), or for 3 d (b). **(c)** Namalwa cells were incubated for 1 d in the presence of GaN as indicated, followed by ^{3}H-thymidine uptake for 12 h. (**d**) Namalwa cells were expanded in the presence of GaN as indicated, and enumerated daily.

Primer sequences for genotyping of mice

Primers	Target	Sequences	Product size (bp)
Eμ-myc for<br>*Eμ-myc rev*	*Eμ-myc*transgene	cagctggcgtaatagcgaagag<br>ctgtgactggtgagtactcaacc	850
Nfatc1flox for<br>*Nfatc1flox rev*	5' *NFATc1 flx* site	cctatttaaacacctccctgcg<br>ccatctctctgaccaacagaagccag	425 (flx)<br>320 (wt)
mb1-cre for<br>*mb1-cre rev*	*mb-1 cre* knock-in	acctctgatgaagtcaggaagaac<br>ggagatgtccttcactctgattct	500
mb1-wt for<br>*mb1-wt rev*	*mb-1 wt* allele	ctgcgggtagaagggggt<br>ccttgcgaggtcagggagcc	400
